# Activation in Context: Differential Conclusions Drawn from Cross-Sectional and Longitudinal Analyses of Adolescents’ Cognitive Control-Related Neural Activity

**DOI:** 10.3389/fnhum.2017.00141

**Published:** 2017-03-24

**Authors:** Ethan M. McCormick, Yang Qu, Eva H. Telzer

**Affiliations:** ^1^Department of Psychology and Neuroscience, University of North Carolina at Chapel HillChapel Hill, NC, USA; ^2^Department of Psychology, Stanford University, StanfordCA, USA

**Keywords:** longitudinal studies, cross-sectional studies, risk-taking, cognitive control, adolescence, fMRI

## Abstract

Although immature cognitive control, subserved by late-developing prefrontal regions, has been proposed to underlie increased risk taking during adolescence, it remains unclear what patterns of PFC activation represent mature brain states: more or less activation? One challenge to drawing cogent conclusions from extant work stems from its reliance on single-time point neuroimaging and cross-sectional comparisons, which are ill-suited for assessing the complex changes that characterize adolescence. This necessitates longitudinal fMRI work to track within-subject changes in PFC function and links to risk-taking behavior, which can serve as an external marker for maturation of neural systems involved in cognitive control. In the current study, 20 healthy adolescents (13 males) completed a go/nogo task during two fMRI scans, once at age 14 years and again at age 15 years. We found that the association between cognitive control-related VLPFC activation and risk-taking behavior reversed when examining wave 1 (W1) versus longitudinal change (W2 > W1) and wave 2 (W2) in neural activation, such that increased VLPFC activation at W1 was associated with *lower* risk taking, whereas longitudinal increases in cognitive control-related VLPFC activation as well as heightened VLPFC activation at W2 were associated with *greater* risk taking. Several steps were taken to disentangle potential alternative accounts that might explain these disparate results across time. Findings highlight the necessity of considering brain-behavior relationships in the context of ongoing developmental changes and suggests that using neuroimaging data at a single time point to predict behavioral changes can introduce interpretation errors when failing to account for changes in neural trajectories.

## Introduction

Teenagers often display a marked lack of the cognitive control necessary to make adaptive decisions, contributing to a steep increase in risk taking during adolescence (Steinberg, 2010). These increases in risky behaviors are thought to reflect, in part, an immature cognitive control system that is unable to effectively regulate the changing physiological and psychosocial influences that occur during this developmental period (Luna et al., 2010; Somerville and Casey, 2010; Somerville et al., 2011). Although immature cognitive control capacities, subserved by a relatively late-developing prefrontal cortex (PFC), have been proposed to underlie this increased risk taking in adolescence (Somerville and Casey, 2010; Somerville et al., 2011; Spear, 2013), no clear picture exists of what patterns of activation represent “immature” or “mature” brain states, with studies finding both increased and decreased activation in adolescents compared with adults. Prior research has largely obtained neuroimaging data at one time point, which may be particularly problematic when studying adolescence, a time of significant neural changes that do not occur simultaneously for all individuals. Instead, longitudinal repeated measures are necessary to track changes in PFC development within individuals as well as links to risk-taking behavior over time.

Cross-sectional studies comparing children, adolescents, and adults have shown mixed patterns of activation in overlapping regions of frontoparietal networks associated with cognitive control (see Crone and Dahl, 2012 for review). Some studies have found that adolescents have reduced activation in ventral and dorsal prefrontal regions compared to adults (Adleman et al., 2002; Bunge et al., 2002; Rubia et al., 2006, 2007; McRae et al., 2012), whereas other studies have found increased activation among adolescents in these same regions (Durston et al., 2002, 2006; Booth et al., 2003; Cohen et al., 2010). Still others have reported both age-related increases and decreases in prefrontal regions from childhood to adulthood (Tamm et al., 2002; Marsh et al., 2006) as well as non-linear changes across development (Van Leijenhorst et al., 2010).

These discrepant results may be due to methodological limitations of cross-sectional designs, which may obscure the relationship between brain and behavior across adolescence. For example, when considering developmental trajectories where all individuals experience change but not necessarily in the same direction or at the same rate, cross sectional designs can lead to results that contradict one another (Kraemer et al., 2000; Maxwell and Cole, 2007). Given the wide variation in the timing and rate of puberty (Palmert and Boepple, 2001; Parent et al., 2003), adolescence may be a period of development which is especially difficult to fully characterize only using cross-sectional designs because individuals of the same chronological age may be in very different places of their individual developmental trajectory. A second limitation of prior cross-sectional studies is the large variability in ages used to represent children, adolescents, and adults. Wide age-ranges for developmental categories can obscure differences that exist within-category, which, given the variability between an average 13 and 18-year-old (Giedd et al., 1999; Spear, 2000), is particularly problematic when studying adolescents. Moreover, by clustering these individuals for comparison with adults, non-linear changes across adolescence may be obscured. These concerns make it difficult to draw cogent conclusions about developmental differences in neural activation related to cognitive control from the largely cross-sectional, extant literature.

In order to address these concerns and draw meaningful conclusions about adolescence, both in terms of cognitive control and general neuro-behavioral development, longitudinal studies are needed to help clarify changes that occur across development by examining individual trajectories across time. Longitudinal studies offer the advantage of removing between-subject variability, instead using the individual as their own baseline for comparison (Louis et al., 1986). Additionally, longitudinal analyses do not make assumptions about the stability of brain-behavior relationships and are particularly well suited to detecting developmental transitions (Kraemer et al., 2000). Finally, in order to fully understand how changes in PFC activation contribute to adolescent risk taking, it is essential to link observed patterns of neural activation with changes in real-world behavior across development (Berkman and Falk, 2013). Because few studies examine the links between changes in neural development and changes in behavior, it remains unclear what neural patterns constitute immaturity – more or less activation (Pfeifer and Allen, 2012). Longitudinal studies may be able to clarify the relationships between activation and behavior, as well as changes in these relationships over time.

In the current study, we followed adolescents over one year to examine how developmental changes in cognitive control-related neural activation predict changes in risk-taking behavior. We used risk-taking as an externally relevant behavioral marker of maturity, building off work that shows that longitudinal decreases in regulatory neural activity is reflected in reduced risky behavior (Qu et al., 2015). In an attempt to reduce noise caused by differences in age among individuals sampled, we recruited a sample of 8^th^ graders who were all 14-years-old at the first wave, and followed them for one year. This is appears to be a period of developmental change for adolescents in terms of structural brain development (Shaw et al., 2008), performance on cognitive control tasks (Luna, 2009), and performance-related neural activation during cognitive control (Koolschijn et al., 2011; Crone and Elzinga, 2015). In light of these changes, brain-behavior relationships may alter substantially as adolescents go through this transition. Given the seemingly contradictory results of prior neuroimaging research (i.e., is more or less activation indicative of a mature neural response? see Crone and Dahl, 2012), we tested whether using a snapshot of neural activation or longitudinal changes in PFC activation in a cognitive control context differentially predict changes in risk-taking behavior.

## Materials and Methods

### Participants

Twenty healthy adolescents participated in the current study at two waves, once in the 8^th^ grade and again in the 9^th^ grade (13 males). At Wave 1 (W1) all adolescents were 14 years old (*M* = 14.39 years, *SD* = 0.34), and at Wave 2 (W2) all adolescents were 15 years old (*M* = 15.20, *SD* = 0.31). At each wave, participants completed an fMRI scan and self-report measures of their risk-taking behavior. An additional three adolescents participated but are not included in analyses (one participant had excessive inter-slice head movement (>2.0 mm), and two participants did not provide self-report data at W1). Adolescent participants provided written assent and parents provided written consent in accordance with the policies of the University’s Institutional Review Board.

### Materials and Procedure

#### Cognitive Control Task

Participants completed a Go-NoGo (GNG) task, which measures behavioral and neural markers of cognitive control. Participants were presented with brief (500 ms) trials in which they saw a single letter and were instructed to press a button to all letters (go trials) with the exception of X (nogo trials). Xs were presented on 25% of the trials. Thus, participants developed a pre-potent response to press during go trials but had to inhibit during no-go trials. Each trial was separated by a fixation period that was jittered with a gamma distribution (*M* = 1000 ms). Participants completed the task four times across four separate blocks. Each block of the task consisted of 80-trials; comprising 60 go and 20 nogo trials. Each block was separated by a 60 s rest period. Effective cognitive control was measured via successfully inhibiting the button press on no-go trials.

#### Adolescent Risk Taking

At both W1 and W2, adolescents reported on their risk-taking behavior using a modified version of the Adolescent Risk-Taking Scale (Alexander et al., 1990; Telzer et al., 2013). Participants completed 12 questions indicating how frequently (1 = *Never* to 4 = *Many Times*) they engaged in a variety of risky behaviors (e.g., “I have gotten high or drunk at a party,” and “I have slipped out at night while my parents thought I was asleep.”). The scale had good internal reliability at both waves (Cronbach’s α: W1 = 0.76; W2 = 0.89). To examine change in risk taking across the two sessions, we computed a difference score representing W2 scores minus W1 scores.

#### Pubertal Development

At both W1 and W2, adolescents self-reported on their level of pubertal maturation using the Peterson Pubertal Developmental Scale (PDS; Petersen et al., 1988). Questions on the PDS assess pubertal development along a number of dimensions including growth, the appearance of body hair, and changes in the skin. Male-specific questions address changes in voice and facial hair, while female-specific questions address breast development and menarche. Participants respond on a four-point scale for each question with (1) indicating that development has not begun, (2) indicating that development has barely begun, (3) indicating that development has substantially begun, and (4) indicating that development is or appears to be complete.

### fMRI Data Acquisition

Imaging data were collected using a 3 Tesla Siemens Trio MRI scanner. The GNG task included T2^∗^-weighted echoplanar images (EPI) [slice thickness = 3mm; 38 slices; TR = 2 s; matrix = 92x92; FOV = 230 mm; voxel size 2.5 mm × 2.5 mm × 3mm]. Structural scans consisted of a T2 weighted, matched-bandwidth (MBW), high-resolution, anatomical scan (TR = 4 s; TE = 64 ms; FOV = 230; matrix = 192 mm × 192mm; slice thickness = 3 mm; 38 slices) and a T1^∗^ magnetization-prepared rapid acquisition gradient echo (MPRAGE; TR = 1.9 s; TE = 2.3 ms; FOV = 230; matrix = 256 × 256; sagittal plane; slice thickness = 1 mm; 192 slices). The orientation for the MBW and EPI scans was oblique axial in order to maximize brain coverage.

#### fMRI Data Preprocessing and Analysis

Data were preprocessed and analyzed using Statistical Parametric Mapping (SPM8; Wellcome Department of Cognitive Neurology, Institute of Neurology, London, UK) software package. Preprocessing was conducted separately for the W1 and W2 scans, which included spatial realignment to correct for head motion (no participant exceeded 1mm of maximum image-to-image motion in any direction), and coregistration with the high-resolution T1^∗^ MPRAGE structural scan, which was subsequently segmented into gray matter, white matter, and cerebrospinal fluid. The transformation matrix used to normalize the MPRAGE images was then applied to the MBW and functional images in order to transform them into the standard stereotactic space defined by the Montreal Neurological Institute and the International Consortium for Brain Mapping. Normalized functional images were smoothed using an 8mm Gaussian kernel, full-width-at-half maximum, to increase the signal-to-noise-ratio. The general linear model in SPM8 was used in order to perform statistical analyses, convolving each trial with a canonical hemodynamic response function. High-pass temporal filtering (cutoff 128 s) was applied to remove low-frequency drift across the time series. Serial autocorrelations were estimated with a restricted maximum likelihood algorithm using an autoregressive model order of 1.

In each participant’s first level model, the preprocessed W1 and W2 scans were concatenated. The task was modeled as an event-related design, with a trial duration of 500ms. In each participant’s fixed-effects model, a general linear model (GLM) was created for each regressor of interest to separate the different events, including successful go trials, successful no-go trials, false alarms (i.e., pressing on no-go trials), and misses (i.e., inhibiting the button response on go trials). These regressors were modeled separately for W1 and W2. Null events consisted of the jittered inter-trial fixation periods plus the one minute rest period between blocks and were not explicitly modeled therefore constituting the implicit baseline. In order to examine linear changes in BOLD signal within the task session, a parametric modulator was included for nogo trials. Trials were linearly weighted, such that trials during the first block were weighted with a 0 and trials in the final block with a 3. By modeling this parametric regressor, we were able to examine linear increases or decreases in BOLD response across the task blocks. To examine longitudinal changes in neural reactivity, contrasts between W1 and W2 were computed at the individual level.

Random effects, group-level analyses were performed on all individual subject contrasts using GLMFlex. GLMFlex corrects for variance-covariance inequality, partitions error terms, removes outliers and sudden activation changes in the brain, and analyzes all voxels containing data^1^. In the current study, all group-level analyses focused on trials where participants successfully inhibited their responses (nogo), as our primary goal was to examine neural activation supporting changes in effective cognitive control. In order to examine how changes in neural activation covaried with changes in self-reported risk-taking behavior, whole-brain regression analyses were conducted by entering changes in risk-taking (i.e., difference score between risk taking at W2–W1) as a regressor.

Correction for multiple comparisons was run using a Monte Carlo simulation through 3dClustSim from the AFNI software package (Ward, 2000, updated April 2016) using the group-level brain mask. The simulation resulted in a voxel-wise threshold of *p* < 0.005 and a minimum cluster size of 68 voxels for the whole brain, corresponding to *p* < 0.05 corrected. In order to plot significant effects, parameter estimates of signal intensity were extracted from the clusters using the MarsBar toolbox in SPM. Because we used a parametric modulator, parameter estimates represent the slope of linear change across weighted trials (i.e., from block 1 to block 4), such that a positive value indicates within session linear increases in activation, a negative value indicates within session linear decreases in activation, and a value of 0 indicates stability in neural activation across the four blocks of the task. Parametric weights were applied across the four blocks in the same manner across individuals and waves for consistency (e.g. block 1 = 0, block 2 = 1, etc.). As such, all group-level whole-brain analyses are computed on the contrasts representing the main effect of block.

## Results

### Behavioral Results

#### Behavioral Performance during Cognitive Control

Participants committed moderate levels of false alarms on average across blocks at both waves (W1: *M* = 27.59%, *SD* = 11.62, *range* = 3.75-47.5%; W2: *M* = 26.72%, *SD* = 14.40, *range* = 1.25–45%). In order to test for effective behavioral inhibition during cognitive control, we examined false alarm rates (i.e., failed inhibition during the go/nogo task) at W1 and W2. We conducted a 4 (block: 1–4) × 2 (wave: W1 and W2) way repeated measures analysis of variance. We found a significant main effect of block on false alarm rate [*F*(1,19) = 13.24, *p* = 0.002, *d* = 0.41] however, no main effect of wave [*F*(1,19) = 0.004, *p* = 0.95, *d* < 0.001] or a wave × block interaction [*F*(1,19) = 0.10, *p* = 0.75, *d* = 0.005]. Adolescents showed increasing false alarms over successive blocks, indicating poorer performance within the task session; however, this effect was similar at both W1 and W2. Adolescent also reported low levels of risk-taking behavior on average [W1: *M* = 1.35, *SD* = 0.32, *range* = 1–2.25; W2: *M* = 1.44, *SD* = 0.50, *range* = 1–2.75], and as a group showed no change in risk taking between W1 and W2 [*t*(19) = –1.36, *p* = 0.19]. Self-reported risk taking at W1, W2, or longitudinal changes in risk taking (W2–W1) were not associated with false alarm rates (within task session or across waves).

### fMRI Results

#### Neural Activation at W1 and Links to Changes in Risk Taking

**FIGURE 1:**
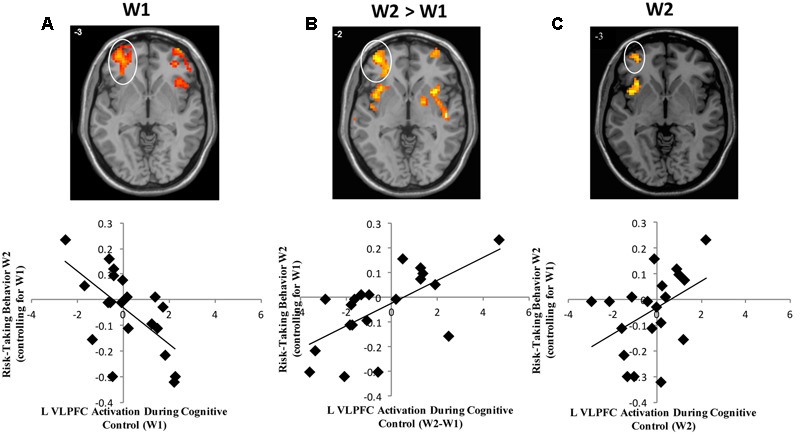
**(A)** Heightened VLPFC activation at W1 is associated with declines in risk-taking behavior. **(B)** Longitudinal declines in VLPFC activation are associated with declines in risk-taking behavior. **(C)** Lower VLPFC activation at W2 is associated with declines in risk-taking behavior.

In whole brain analyses, we first examined the main effect of neural activation across the task blocks during successful Nogo trials at W1. As shown in **Table 1**, adolescents showed decreased activation in the right parietal lobe and bilateral calcarine gyri. To examine brain-behavior links, we next examined how brain activation at W1 was associated with risk taking at W2. In whole brain regression analyses, we regressed risk-taking behavior at W2 (controlling for W1) onto neural activation during successful Nogo trials across the blocks at W1. Results indicate a negative correlation in the bilateral ventrolateral prefrontal cortex (VLPFC; **Table 2**). For descriptive purposes, we extracted parameter estimates of signal intensity from the VLPFC cluster and plotted this activation with adolescents’ risk-taking behavior. As shown in **Figure 1A**, greater activation in VLPFC activation at W1 was associated with lower risk-taking behavior at W2, suggesting an adaptive role of greater VLPFC activation.

**Table 1:** Neural regions showing a main effect of task block.

Anatomical Region	±	BA	*x*	*y*	*z*	*t*	*k*
*W1 Activation*							
Calcarine Gyrus	–	17	–6	–91	–5	5.20	712
R Inferior Parietal Lobule^a^	–	39	45	–49	52	6.27	86
R Precuneus^a^	–	7	18	–46	52	3.74	
*Change in Activation (W2–W1)*							
R Supramarginal Gyrus	+	40	66	–31	28	4.32	88

*L and R refer to left and right hemispheres; + and – refer to positive or negative effect; BA refers to Brodmann Area of peak voxel; *k* refers to the number of voxels in each significant cluster; *t* refers to peak activation level in each cluster; *x, y*, and *z* refer to MNI coordinates. ^a^indicate that peak voxels are part of a contiguous cluster.*

**Table 2:** Neural regions that correlated with changes in risk taking during successful nogo trials.

Anatomical Region	±	BA	*x*	*y*	*z*	*t*	*k*
*W1 Activation*							
L Ventrolateral Prefrontal Cortex	–	10	–36	53	–2	5.14	209
R Ventrolateral Prefrontal Cortex	–	10	39	62	–5	4.54	73
Cerebellum	–		0	–52	–41	7.18	231
*Change in Activation (W2–W1)*							
L Ventrolateral Prefrontal Cortex	+	10	–39	53	–2	6.59	286
R Insula	+		36	14	–2	5.16	226
R Superior Medial Gyrus	+	8	3	29	52	4.15	80
Cerebellum	+		0	–52	–41	6.52	227
*W2 Activation*							
L Ventrolateral Prefrontal Cortex^∗^	+	10	–33	50	–2	3.64	31
L Insula^∗^	+		–36	20	1	4.43	57

*L and R refer to left and right hemispheres; + and – refer to positive or negative association; BA refers to Brodmann Area of peak voxel; *k* refers to the number of voxels in each significant cluster; *t* refers to peak activation level in each cluster; *x, y*, and *z* refer to MNI coordinates. ^∗^Sub-threshold cluster (*k*) sizes, but included for exploratory analyses.*

#### Longitudinal Changes in Neural Activation and Links to Changes in Risk Taking

To examine longitudinal changes in neural activation across waves, we computed a whole-brain *t*-test to examine differences in activation between W2 and W1 for successful Nogo trials. We found longitudinal changes in the right inferior parietal lobe such that participants showed increased activation across blocks at W2 relative to W1 (**Table 1**). Next, we examined how within-subject (e.g., longitudinal) change in brain activation was related to longitudinal changes in risk-taking behavior. We conducted whole-brain regression analyses in which we regressed adolescent risk taking at W2 (controlling for W1) onto changes in neural activation during successful Nogo trials (Nogo W2 > Nogo W1). Results indicate a positive correlation in the bilateral VLPFC in nearly identical regions identified by the cross-sectional, W1 effects, but in the opposite direction, such that adolescents who showed longitudinal declines in VLPFC activation from W1 to W2 showed lower risk taking (**Table 2** and **Figure 1B**). Interestingly, activation in the same neural region showed a completely opposite and seemingly contradictory relationship with risk taking depending on whether activation was measured at a single time-point or longitudinally.

### Exploring Disparate Results

**FIGURE 2:**
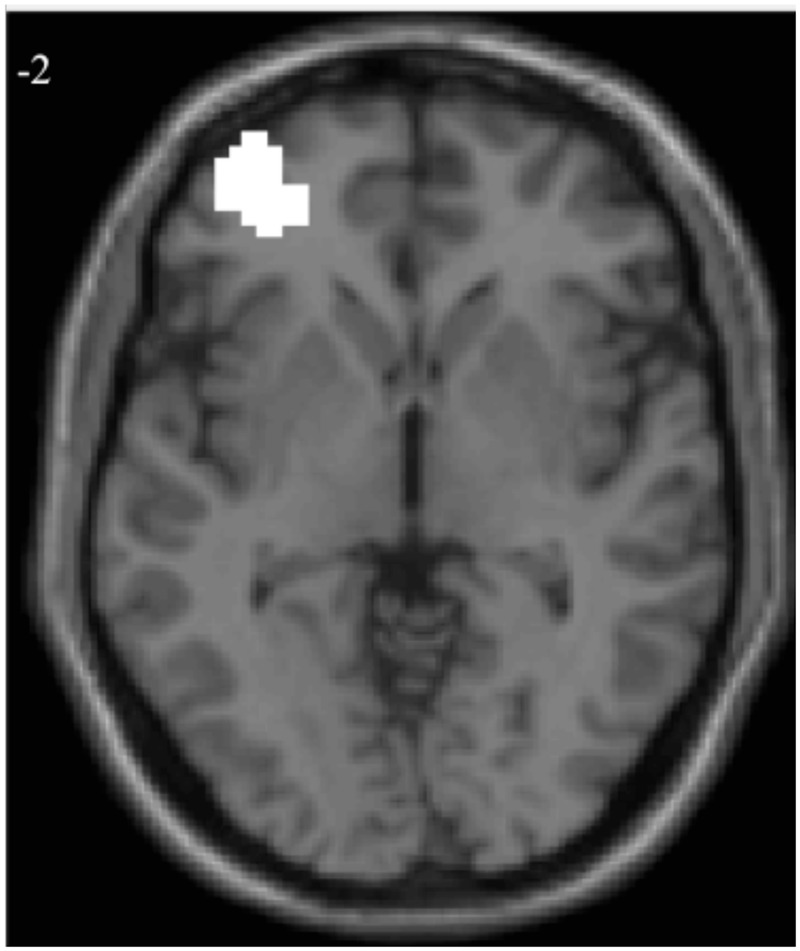
**Overlapping voxels for regressions of adolescent risk taking with W1 and W2 > W1 neural activation**.

Given that VLPFC activation when examined at W1 predicted lower risk taking, whereas changes in VLPFC activation from W1 to W2 predicted greater risk taking, we wanted to see if we could disentangle how examining VLFPC activity at W1 versus W2-W1 could change the conclusions drawn about brain-behaviors associations. We explored two potential explanations: the results are a statistical fluke (e.g., regression to the mean), or we are capturing a meaningful developmental transition. For exploratory purposes, we extracted parameter estimates of signal intensity for all neural analyses (i.e., W1, W2, and W2 > W1 contrasts) from the mask constructed from the overlap between the significant VLPFC clusters found in the W1 and the W2 > W1 contrasts (**Figure 2**). All statistical analyses reported below were run in SPSS using the extracted parameter estimates of signal intensity from this cluster.

#### Neural Activation at W2 and Links to Changes in Risk Taking

Because we saw an inversion of brain-behavior relationships when examining W1 and W2 > W1 risk taking effects with the VLPFC, we examined W2 neural activation alone. To examine how neural activation at W2 is associated with risk-taking behavior, we conducted a whole-brain regression analysis, in which we regressed W2 risk taking (controlling for W1 risk taking) onto brain activation at W2. We found a positive association between risk taking and activity in a small, cluster-level uncorrected (*k* = 30) group of voxels in the left VLPFC (see **Table 2**). As shown in **Figure 1C**, greater activation in VLPFC at W2 was associated with greater risk taking at W2 confirming the longitudinal neuroimaging results. These results seem to argue against a regression to the mean hypothesis, and instead suggest that the positive change in VLPFC activation is not driven completely by change from a negative association between VLPFC activation and W2 risk taking (controlling for W1) to a flat relationship at W2. Rather, the strong positive change seen in the W2 > W1 contrast reflects a significant change from a negative to a positive association.

#### Plotting Individual Change

**FIGURE 3:**
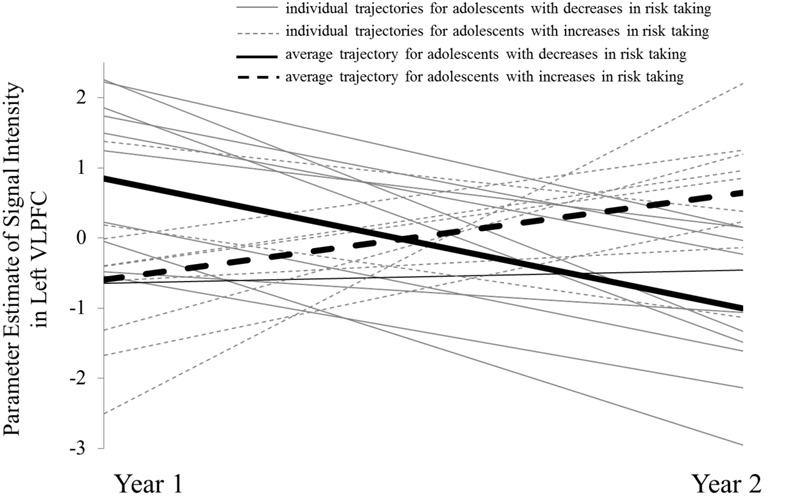
**Individual trajectories of VLPFC activation for adolescents who show decreases in risk taking (solid line) and increases in risk taking (dashed line)**.

Next, we extracted parameter estimates of signal intensity from the left VLPFC cluster separately from W1 and W2, and plotted individual trajectories for each subject’s neural activation at W1 and W2 (**Figure 3**). For descriptive purposes, we divided the sample into adolescents who showed increases in risk taking across the year (dashed lines with the average slope depicted with the thick dashed line) and adolescents who showed decreases in risk taking (solid lines with the average slope depicted with the thick solid line). As evidenced by the initial intercept (i.e., W1 activation), adolescents who reported subsequent decreases in risk taking were likely to have higher average activation at W1 (thick solid line) compared to adolescents who increased in risk taking [*t*(18) = 2.80, *p* = 0.012], a pattern that reversed at W2 [*t*(18) = –3.70, *p* = 0.002]. Moreover, adolescents who showed decreases in risk taking showed significant declines in VLPFC activation [*b* = 1.24, *t*(10) = 5.41, *p <* 0.001] while adolescents who showed increases in risk taking showed trending increases in VLPFC activation [*b* = –1.85, *t*(8) = 2.05, *p* = 0.07]. As the solid and dashed lines show, these trajectories caused the average activation of each group to cross one another, and this crossover resulted in the flip in relationship between brain and behavior seen in the whole-brain regression analyses. If our results were due to regression to the mean, we would expect that neural trajectories would converge instead of cross over and flip in direction.

#### Mediation Analysis

**FIGURE 4:**
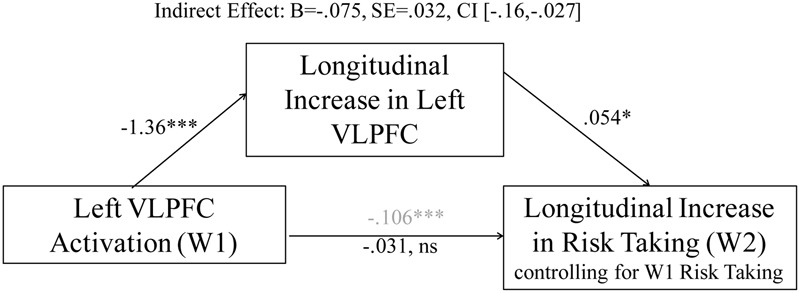
**The relationship between left VLPFC activity at W1 and changes in risk taking is mediated by longitudinal changes in left VLPFC activation.** ^∗^*p* < 0.05, ^∗∗∗^*p* < 0.0001.

To further examine how W1 and W2 > W1 VLPFC activation are differentially associated with risk-taking behavior, we conducted mediation analyses in which we examined the indirect effect of W1 VLPFC activation on risk-taking behavior through longitudinal changes in VLPFC activation. This method allows us to examine whether (1) patterns of brain activation at W1 contribute to changes in brain activation over time (i.e., do adolescents who show high VLPFC activation at W1 those who then show declines in VLPFC activation over the year), and (2) whether longitudinal changes in VLPFC activation explain the original relationship between VLPFC activation at W1 and risk-taking behavior. Using the methods outlined by Hayes (Hayes, 2013), we used 1,000 bootstrap samples to calculate the magnitude of the indirect effect using a bias-corrected confidence interval (CI). As shown in **Figure 4**, heightened VLPFC activation at W1 was associated with longitudinal declines in VLPFC activation from W1 to W2 as well as lower risk taking at W2. The direct path becomes non-significant when accounting for longitudinal change in VLPFC activation, and the indirect path from W1 VLPFC activation to risk taking through changes in VLPFC activation is significant, consistent with statistically significant mediation. These results further suggest that the flip in association between VLPFC activation and changes in risk taking behavior are likely to reflect developmental changes.

#### Concurrent and Longitudinal Associations

**FIGURE 5:**
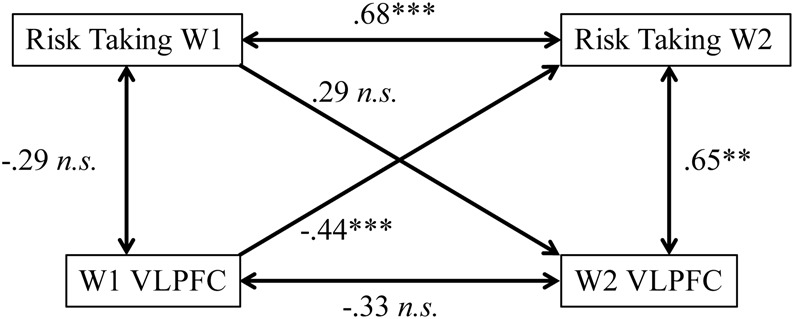
**Correlations between variables at W1 and W2.** While W1 VLPFC activity predicts W2 risk taking controlling for W1 risk taking, the reciprocal path of W1 Risk Taking to W2 VLFPC activation controlling for W1 activity is not significant. Zero-order correlations are indicated with double-headed arrows, while regression analyses controlling for both W1 variables are indicated with single-headed arrows. ^∗∗^*p* < 0.005, ^∗∗∗^*p* < 0.0001.

Finally, we wanted to test how VLPFC activity and risk-taking behavior are associated with one another within a single time point and across the year. To test concurrent associations, we ran first-order correlations between VLPFC activation and risk-taking behavior at each wave. We found that at W1, risk taking and VLPFC activity are not related (*r* = –0.29, *p* = 0.21); however, at W2, there is a moderately strong positive association between risk-taking behavior and VLPFC activation (*r* = 0.65, *p* = 0.002; **Figure 5**). Because the association between brain and behavior across time appears to be changing, we ran a Fisher’s r-to-z transformation to test for differences between associations at W1 and W2. Results indicated that there is a significant change in the association between risk-taking behavior and VLPFC activation between W1 and W2 [*z* = –3.13, *p*_(one-tailedtest)_ < 0.001]. To test longitudinal associations, we performed linear regression analyses with risk-taking behavior and VLPFC activation at W1 simultaneously to test for associations with risk taking behavior at W2. Results showed that both W1 risk taking (β = 0.68, *p* < 0.001) and W1 VLPFC activity (β = –0.44, *p* = 0.001) were significantly associated with W2 risk taking. In contrast, when we entered W1 risk-taking and W1 VLPFC to test for associations with W2 VLPFC activation, results showed that neither risk-taking behavior (β = 0.29, *p* = 0.21) nor W1 VLPFC (*β* = –0.33, *p* = 0.15) were significantly related (**Figure 5**). Importantly, these results show that both risk-taking behavior and brain activation at W1 are associated with risk-taking behavior one year later, but risk-taking behavior at W1 is not associated with brain activation one year later. While not confirmatory, these findings together suggest a potential role for the VLPFC in predicting risk-taking behavior changes.

### Pubertal Development and Gender Differences

Finally, we wanted to examine whether pubertal maturation or gender were associated with our constructs of interest. Adolescents showed higher levels of pubertal development at W2 (*M* = 3.20, *SD* = 0.29, *range* = 2.40–4.00) than W1 (*M* = 2.95, *SD* = 0.60, *range* = 1.60–4.00), *t*(19) = 3.39, *p* = 0.003). Age and pubertal development were not significantly related at W1 or W2 (W1: *r* = 0.322, *p* = 0.17; W2: *r* = –0.04, *p* = 0.86), likely due to the very restricted age range. Female participants showed marginally more pubertal development at W1 than males [*t*(18) = –1.97, *p* = 0.065] and significantly more pubertal development at W2 [*t*(18) = –2.99, *p* = 0.008], although there was no significant gender difference in changes in pubertal development across the year [*t*(18 = 0.06, *p* = 0.95]. Pubertal maturation at W1 was associated with greater risk-taking behavior at W1 (*r* = 0.60, *p* = 0.005), but pubertal maturation at W1 did not predict W2 risk-taking behavior (controlling for W1 risk taking). Pubertal maturation at W2 was marginally associated with risk-taking behavior at W2 (*r* = 0.44, *p* = 0.053). Pubertal maturation at W1 was not associated with VLPFC activation at W1, W2, or W2 > W1. In terms of gender, males and females did not differ in VLPFC activation at W1 [*t*(18) = 0.96, *p* = 0.35] or W2 [*t*(18) = –0.63, *p* = 0.54] nor in risk-taking behavior at W1 [*t*(18) = –1.08, *p* = 0.30] or W2 [*t*(18) = –1.20, *p* = 0.24]. Finally, we re-ran all primary analyses of interest, controlling for age, and gender, and either pubertal development at W1 or the change in pubertal development. Controlling for these variables in our mediation model or including these variables as additional controls in our whole brain regression analyses, did not impact any of the effects we observed.

## Discussion

Prior research examining developmental differences in cognitive control-related brain function have produced mixed and contradictory results (Crone and Dahl, 2012). These contradictions have made it difficult to draw meaningful conclusions from the current literature. Prior work has relied almost entirely on cross-sectional measurements of the brain (for exceptions see Koolschijn et al., 2011; Ordaz et al., 2013; Braams et al., 2015; Paulsen et al., 2015), which can fail to capture important aspects of development. This is especially true in adolescence when individuals do not necessarily experience change at the same rate or in the same direction (Kraemer et al., 2000). Using longitudinal fMRI techniques, we found that cross-sectional approaches can result in apparently contradictory conclusions concerning relationships between brain and behavior. The reversal in the association between cognitive control-related VLPFC activation and changes in risk taking observed in the current study may reflect changing brain-behavior relationships across this transitional period of development. While further research will be required to test these hypotheses, it may be that divergent patterns of maturation in VLPFC regions result in these apparently contradictory findings. Delayed maturation of neural regions involved in cognitive control may limit adolescents’ ability to regulate their behavior, resulting in increases in risky behavior.

Our findings show that at age 14 years, increased activation in the ventrolateral prefrontal cortex during cognitive control was associated with less risk-taking behavior one year later. However, longitudinal neural trajectories from ages 14 to 15 show the opposite pattern, such that declines in activation during cognitive control were related to declines in risk taking. We explored this apparent discrepancy in a number of ways to assess whether our results may be driven by spurious statistical effects (e.g., regression to the mean) or may represent a meaningful development transition. First, we examined the association between risk-taking behavior and W2 neural activation on its own. We found, in contrast with W1 effects, that W2 VLPFC activation was positively associated with W2 risk-taking behavior, indicating a significant flip in the association between brain and behavior. We also plotted individual neural trajectories in order to examine the possibility that individuals are converging across the year in terms of brain activation. Rather than converging, these trajectories suggested that the adolescents who showed increased W2 risk taking versus decreased W2 risk taking had opposite patterns of neural change, crossing one another between 14- and 15-years of age. Thirdly, we performed mediation analyses to test whether longitudinal brain changes explained the association between W1 VLPFC activation and W2 risk-taking behavior. We found that longitudinal changes in VLPFC activation explained, in part, the relationship between W1 VLPFC activation and W2 risk-taking behavior. These results highlight the importance of using neural change to predict behavior during development. Fourth, using regression analyses to predict W2 brain and behavior show that while W1 VLPFC activation predicts W2 risk-taking (controlling for W1 risk taking), W1 risk taking does not predict W2 neural activation. Additionally, the relationship between VLPFC activation and risk-taking behavior changes from a negative (albeit non-significant) association to a moderately strong positive relationship, further bolstering the conclusion that this period of development involves significant changes in brain-behavior relationships.

The seemingly contradictory relationship between neural activation and risk taking may suggest that between ages 14 and 15, the relationship between neural activation and behavior reverses. This pattern is consistent with an inflection point in neural maturation related to cognitive control seen around 14 years of age (Bunge and Wright, 2007; Luna, 2009; Crone and Elzinga, 2015). While previous literature has not utilized the Go/No-Go task specifically, other behavioral inhibition paradigms (e.g., see Luna, 2009) recruit similar regions of the VLPFC, suggesting that this period of development is a time of transition in cognitive control-related neural processes. Although increases in activation leading up to this inflection point may relate to more-optimal outcomes, further increases are disadvantageous such that youths who show additional increases in PFC activation display suboptimal decision making outcomes, whereas adolescents who reach the peak and show subsequent declines in PFC activation show declines in risk taking. This may imply that during earlier development, increases in activation represent skill acquisition. However, after stabilizing at age 14, activational decreases during cognitive control may allow PFC regions to be less energetically wasteful, diverting resources to other regions (Kelly and Garavan, 2005). Because we constrained the age of participants in order to reduce within-wave age-related variability, the specificity of our results to 14-15 year olds reduces our ability to generalize our findings across adolescence. Additional waves are necessary to extend these findings and to confirm the hypothesis of transitions in cognitive control-related neural regions; however, results thus far suggest that longitudinal approaches can both reveal neurodevelopmental effects invisible to cross-sectional studies as well as resolve disparate cross-sectional findings.

To further clarify our results, we wanted to see if we could account for the differential associations between VLPFC activation and risk-taking behavior by controlling for potential maturation effects. Previous research has shown links between pubertal maturation and increases in risk taking that are independent of chronological age (Costello et al., 2007; Steinberg et al., 2008; Downing and Bellis, 2009; de Water et al., 2013), although these effects are not always consistent across genders (e.g., Steinberg et al., 2008; de Water et al., 2013). To address these concerns, we tested for both gender and pubertal maturation effects in our analyses. Although pubertal development was associated with some measures of risk taking, pubertal development was not associated with neural activation in the VLPFC, and gender was not associated with any variables of interest. Additionally, controlling for pubertal development and gender did not impact the results of our mediation or our regression models. Even though other forms of maturation cannot be ruled out as accounting for the effects we observe, when taken together, our findings suggest that adolescents during this period of development may be undergoing a change in how brain and behavior are related to one another in ways that are not easily explained by pubertal development.

Our results demonstrate some of the potential pitfalls of using cross-sectional neural activations in developmental populations to predict behavioral outcomes. Unless the meaning and function of neural activation remains stable across the period of development being studied, this approach can yield mixed results. The high degree of neural changes during adolescence renders this period of development particularly opaque to cross-sectional analysis, further emphasizing the importance of longitudinal work to complement the current literature. In the current study, results suggest that if we had only sampled at age 14 years, we would have arrived at very different conclusions than if we had only sampled at age 15 years. Additionally, if we sampled across this age range, the relative mix of younger and older participants likely would have biased which effect we were likely to find. In order to unpack developmental trajectories in neural development, future longitudinal work should focus on extending our knowledge about neural trajectories. Measuring 12–13 and 16–17 year olds will help to resolve the shape of this transition and implications for adolescents who develop earlier or later than their peers. Furthermore, extending longitudinal examination of these processes can allow us to examine heterogeneous, and potentially non-linear, developmental trajectories across individuals, which two- and even three-wave longitudinal studies have difficulty resolving (Raz et al., 2010; Raznahan et al., 2011). By comparing how changes (i.e., increases or decreases) in activation across adolescence relate to changes in behavior, we can gain a clearer picture of which patterns of activation (i.e., more or less) represent more-mature brain states.

In light of these results, several next steps can help to extend our knowledge concerning the processes of neural maturation, its consequences for behavior, and other possible methodological issues in measuring change across time in adolescence. The first, as alluded to earlier, is to extend the age range under examination. While constraining the age range of adolescence at each wave was helpful in restricting within-wave developmental differences, it limits our ability to say if these contradictory findings are specific to 14–15 year olds. Furthermore, the lack of behavioral associations between behavior on the Go/No-Go task and risky behavior was both a strength and limitation of the current analyses. While the task does not appear to have been able to discriminate significant inter-subject variability in cognitive control, the equitability of behavioral performance suggests that individuals who show differing directions of change across time may be employing the VLPFC differently during behavioral inhibition trials rather than indexing an ability to complete the task. Future work could perhaps use on an adaptive version of the Go/No-Go (e.g., the Stop Signal task) that can both equate the number of successful inhibition trials, as well as better discriminate between different competencies on the task. Finally, future research should specifically explore the hypothesis that the VLPFC is being used differently by different groups of adolescents. If adolescents are using different VLPFC-dependent strategies to employ cognitive control, these individual differences may have important consequences for how successfully adolescents can regulate their behavior in more-challenging contexts. Finally, although we focused on the VLPFC in the current manuscript because of our *a priori* hypothesis about its involvement in cognitive control-related processes, the insula also shows similar (although not as consistent) patterns to the VLPFC across this period of development. Previous work has shown insula involvement in regulatory processes (e.g., Hampshire et al., 2010; Goldenberg et al., 2013), and future work should seek to explore the joint and specific contributions that VLPFC and insula are making to cognitive control processes and their impact on risk-taking behavior.

## Conclusion

Results from the current study reveal inferential pitfalls inherent in cross-sectional neuroimaging work. When predicting adolescent engagement in risk taking using cross-sectional and longitudinal neuroimaging data, we found that these approaches yield completely opposite relationships between neural activation and risk taking. This further suggests that using neuroimaging data at a single time point to predict behavioral changes can introduce serious interpretation errors when failing to account for changes in neural trajectories and highlights the need for longitudinal work to augment the extant literature on neural development, cognitive control, and risk taking in adolescents.

## Ethics Statement

This study was carried out in accordance with the recommendations of the University of Illinois, Institutional Review Board with written informed consent and assent from all subjects. All subjects gave written informed consent and assent in accordance with the Declaration of Helsinki. The protocol was approved by the University of Illinois; Institutional Review Board.

## Author Contributions

ET designed research; ET and YQ performed research; EM and ET analyzed data; EM, YQ, and ET wrote the paper.

## Conflict of Interest Statement

The authors declare that the research was conducted in the absence of any commercial or financial relationships that could be construed as a potential conflict of interest.

## Footnotes

1 http://mrtools.mgh.harvard.edu/index.php/GLM_Flex

## References

[B1] Adleman N. E. Menon V. Blasey C. M. White C. D. Warsofsky I. S. Glover G. H. (2002). A developmental fMRI study of the Stroop color-word task. *Neuroimage* 16 61–75. 10.1006/nimg.2001.1046 11969318

[B2] Alexander C. S. Kim Y. J. Ensminger M. Johnson K. E. Smith B. J. Dolan L. J. (1990). A measure of risk taking for young adolescents: reliability and validity assessments. *J. Youth Adolesc.* 19 559–569. 10.1007/BF01537176 24272744

[B3] Berkman E. T. Falk E. B. (2013). Beyond brain mapping: using neural measures to predict real-world outcomes. *Curr. Dir. Psychol. Sci.* 22 45–50. 10.1177/0963721412469394 24478540 PMC3903296

[B4] Booth J. R. Burman D. D. Meyer J. R. Lei Z. Trommer B. L. Davenport N. D. (2003). Neural development of selective attention and response inhibition. *Neuroimage* 20 737–751. 10.1016/S1053-8119(03)00404-X 14568448

[B5] Braams B. R. van Duijvenvoorde A. C. Peper J. S. Crone E. A. (2015). Longitudinal changes in adolescent risk-taking: a comprehensive study of neural responses to rewards, pubertal development, and risk-taking behavior. *J. Neurosci.* 35 7226–7238. 10.1523/JNEUROSCI.4764-14.2015 25948271 PMC6605271

[B6] Bunge S. A. Dudukovic N. M. Thomason M. E. Vaidya C. J. Gabrieli J. D. E. (2002). Immature frontal lobe contributions to cognitive control in children. *Neuron* 33 301–311. 10.1016/S0896-6273(01)00583-9 11804576 PMC4535916

[B7] Bunge S. A. Wright S. B. (2007). Neurodevelopmental changes in working memory and cognitive control. *Curr. Opin. Neurobiol.* 17 243–250. 10.1016/j.conb.2007.02.005 17321127

[B8] Cohen J. R. Asarnow R. F. Sabb F. W. Bilder R. M. Bookheimer S. Y. Knowlton B. J. (2010). Decoding developmental differences and individual variability in response inhibition through predictive analyses across individuals. *Front. Hum. Neurosci.* 4:47 10.3389/fnhum.2010.00047 PMC2906202 20661296

[B9] Costello E. J. Sung M. Worthman C. Angold A. (2007). Pubertal maturation and the development of alcohol use and abuse. *Drug Alcohol Depend.* 88 S50–S59. 10.1016/j.drugalcdep.2006.12.009 17275214

[B10] Crone E. A. Dahl R. E. (2012). Understanding adolescence as a period of social-affective engagement and goal flexibility. *Nat. Rev. Neurosci.* 13 636–650. 10.1038/nrn3313 22903221

[B11] Crone E. A. Elzinga B. M. (2015). Changing brains: how longitudinal functional magnetic resonance imaging studies can inform us about cognitive and social-affective growth trajectories. *Wiley Interdiscip. Rev. Cogn. Sci.* 6 53–63. 10.1002/wcs.1327 26262928

[B12] de Water E. Braams B. R. Crone E. A. Peper J. S. (2013). Pubertal maturation and sex steroids are related to alcohol use in adolescents. *Horm. Behav.* 63 392–397. 10.1016/j.yhbeh.2012.11.018 23229027

[B13] Downing J. Bellis M. A. (2009). Early pubertal onset and its relationship with sexual risk taking, substance use and anti-social behaviour: a preliminary cross-sectional study. *BMC Public Health* 9:446 10.1186/1471-2458-9-446 PMC3091535 19958543

[B14] Durston S. Davidson M. C. Tottenham N. Galvan A. Spicer J. Fossella J. A. (2006). A shift from diffuse to focal cortical activity with development. *Dev. Sci.* 9 1–8. 10.1111/j.1467-7687.2005.00454.x 16445387

[B15] Durston S. Thomas K. M. Yang Y. Ulug A. M. Zimmerman R. D. Casey B. J. (2002). A neural basis for the development of inhibitory control. *Dev. Sci.* 5 F9–F16. 10.1111/1467-7687.00235

[B16] Goldenberg D. Telzer E. H. Lieberman M. D. Fuligni A. Galván A. (2013). Neural mechanisms of impulse control in sexually risky adolescents. *Dev. Cogn. Neurosci.* 6 23–29. 10.1016/j.dcn.2013.06.002 23835204 PMC3924962

[B17] Giedd J. N. Blumenthal J. Jeffries N. O. Castellanos F. X. Liu H. Zijdenbos A. (1999). Brain development during childhood and adolescence: a longitudinal MRI study. *Nat. Neurosci.* 2 861–863. 10.1038/13158 10491603

[B18] Hampshire A. Chamberlain S. R. Monti M. M. Duncan J. Owen A. M. (2010). The role of the right inferior frontal gyrus: inhibition and attentional control. *Neuroimage* 50 1313–1319. 10.1016/j.neuroimage.2009.12.109 20056157 PMC2845804

[B19] Hayes A. F. (2013). *Introduction to Mediation, Moderation, and Conditional Process Analysis: A Regression-Based Approach.* New York, NY: Guilford Press.

[B20] Kelly A. M. C. Garavan H. (2005). Human functional neuroimaging of brain changes associated with practice. *Cereb. Cortex* 15 1089–1102. 10.1093/cercor/bhi005 15616134

[B21] Koolschijn P. C. M. Schel M. A. de Rooij M. Rombouts S. A. Crone E. A. (2011). A three-year longitudinal functional magnetic resonance imaging study of performance monitoring and test-retest reliability from childhood to early adulthood. *J. Neurosci.* 31 4204–4212. 10.1523/JNEUROSCI.6415-10.2011 21411661 PMC6623527

[B22] Kraemer H. C. Yesavage J. A. Taylor J. L. Kupfer D. (2000). How can we learn about developmental processes from cross-sectional studies, or can we? *Am. J. Psychiatry* 157 163–171. 10671382 10.1176/appi.ajp.157.2.163

[B23] Louis T. A. Robins J. Dockery D. W. Spiro A. Ware J. H. (1986). Explaining discrepancies between longitudinal and cross-sectional models. *J. Chronic Dis.* 39 831–839. 10.1016/0021-9681(86)90085-8 3489727

[B24] Luna B. (2009). Developmental changes in cognitive control through adolescence. *Adv. Child Dev. Behav.* 37 233–278. 10.1016/s0065-2407(09)03706-9 19673164 PMC2782527

[B25] Luna B. Padmanabhan A. O’Hearn K. (2010). What has fMRI told us about the development of cognitive control through adolescence? *Brain Cogn.* 72 101–113. 10.1016/j.bandc.2009.08.005 19765880 PMC2815087

[B26] Marsh R. Zhu H. Schultz R. T. Quackenbush G. Royal J. Skudlarski P. (2006). A developmental fMRI study of self-regulatory control. *Hum. Brain Mapp.* 27 848–863. 10.1002/hbm.20225 16421886 PMC2292452

[B27] Maxwell S. E. Cole D. A. (2007). Bias in cross-sectional analyses of longitudinal mediation. *Psychol. Methods* 12:23 10.1037/1082-989X.12.1.23 17402810

[B28] McRae K. Gross J. J. Weber J. Robertson E. R. Sokol-Hessner P. Ray R. D. (2012). The development of emotion regulation: an fMRI study of cognitive reappraisal in children, adolescents and young adults. *Soc. Cogn. Affect. Neurosci.* 7 11–22. 10.1093/scan/nsr093 22228751 PMC3252634

[B29] Ordaz S. J. Foran W. Velanova K. Luna B. (2013). Longitudinal growth curves of brain function underlying inhibitory control through adolescence. *J. Neurosci.* 33 18109–18124. 10.1523/JNEUROSCI.1741-13.2013 24227721 PMC3828464

[B30] Palmert M. R. Boepple P. A. (2001). Variation in the timing of puberty: clinical spectrum and genetic investigation. *J. Clin. Endocrinol. Metab.* 86 2364–2368. 10.1210/jcem.86.6.7603 11397824

[B31] Parent A. S. Teilmann G. Juul A. Skakkebaek N. E. Toppari J. Bourguignon J. P. (2003). The timing of normal puberty and the age limits of sexual precocity: variations around the world, secular trends, and changes after migration. *Endocr. Rev.* 24 668–693. 10.1210/er.2002-0019 14570750

[B32] Paulsen D. J. Hallquist M. N. Geier C. F. Luna B. (2015). Effects of incentives, age, and behavior on brain activation during inhibitory control: a longitudinal fMRI study. *Deve. Cogn. Neurosci.* 11 105–115. 10.1016/j.dcn.2014.09.003 PMC4323861 25284272

[B33] Petersen A. C. Crockett L. Richards M. Boxer A. (1988). A self-report measure of pubertal status: reliability, validity, and initial norms. *J. Youth Adolesc.* 17 117–133. 10.1007/BF01537962 24277579

[B34] Pfeifer J. H. Allen N. B. (2012). Arrested development? Reconsidering dual-systems models of brain function in adolescence and disorders. *Trends Cogn. Sci.* 16 322–329. 10.1016/j.tics.2012.04.011 22613872 PMC3711850

[B35] Qu Y. Galvan A. Fuligni A. J. Lieberman M. D. Telzer E. H. (2015). Longitudinal changes in prefrontal cortex activation underlie declines in adolescent risk taking. *J. Neurosci.* 35 11308–11314. 10.1523/JNEUROSCI.1553-15.2015 26269638 PMC4532760

[B36] Raz N. Ghisletta P. Rodrigue K. M. Kennedy K. M. Lindenberger U. (2010). Trajectories of brain aging in middle-aged and older adults: regional and individual differences. *Neuroimage* 51 501–511. 10.1016/j.neuroimage.2010.03.020 20298790 PMC2879584

[B37] Raznahan A. Lerch J. P. Lee N. Greenstein D. Wallace G. L. Stockman M. (2011). Patterns of coordinated anatomical change in human cortical development: a longitudinal neuroimaging study of maturational coupling. *Neuron* 72 873–884. 10.1016/j.neuron.2011.09.028 22153381 PMC4870892

[B38] Rubia K. Smith A. B. Taylor E. Brammer M. (2007). Linear age-correlated functional development of right inferior fronto-striato-cerebellar networks during response inhibition and anterior cingulate during error-related processes. *Hum. Brain Mapp.* 28 1163–1177. 10.1002/hbm.20347 17538951 PMC6871440

[B39] Rubia K. Smith A. B. Woolley J. Nosarti C. Heyman I. Taylor E. (2006). Progressive increase of frontostriatal brain activation from childhood to adulthood during event-related tasks of cognitive control. *Hum. Brain Mapp.* 27 973–993. 10.1002/hbm.20237 16683265 PMC6871373

[B40] Shaw P. Kabani N. J. Lerch J. P. Eckstrand K. Lenroot R. Gogtay N. (2008). Neurodevelopmental trajectories of the human cerebral cortex. *J. Neurosci.* 28 3586–3594. 10.1523/JNEUROSCI.5309-07.2008 18385317 PMC6671079

[B41] Somerville L. H. Casey B. J. (2010). Developmental neurobiology of cognitive control and motivational systems. *Curr. Opin. Neurobiol.* 20 236–241. 10.1016/j.conb.2010.01.006 20167473 PMC3014528

[B42] Somerville L. H. Hare T. Casey B. J. (2011). Frontostriatal maturation predicts cognitive control failure to appetitive cues in adolescents. *J. Cogn. Neurosci.* 23 2123–2134. 10.1162/jocn.2010.21572 20809855 PMC3131482

[B43] Spear L. P. (2000). The adolescent brain and age-related behavioral manifestations. *Neurosci. Biobehav. Rev.* 24 417–463. 10.1016/S0149-7634(00)00014-2 10817843

[B44] Spear L. P. (2013). Adolescent neurodevelopment. *J. Adolesc. Health* 52 S7–S13. 10.1016/j.jadohealth.2012.05.006 23332574 PMC3982854

[B45] Steinberg L. (2010). A dual systems model of adolescent risk-taking. *Dev. Psychobiol.* 52 216–224. 10.1002/dev.20445 20213754

[B46] Steinberg L. Albert D. Cauffman E. Banich M. Graham S. Woolard J. (2008). Age differences in sensation seeking and impulsivity as indexed by behavior and self-report: evidence for a dual systems model. *Dev. Psychol.* 44 1764 10.1037/a0012955 18999337

[B47] Tamm L. Menon V. Reiss A. L. (2002). Maturation of brain function associated with response inhibition. *J. Am. Acad. Child Adolesc. Psychiatry* 41 1231–1238. 10.1097/00004583-200210000-00013 12364845

[B48] Telzer E. H. Fuligni A. J. Lieberman M. D. Galván A. (2013). Meaningful family relationships: neurocognitive buffers of adolescent risk taking. *J. Cogn. Neurosci.* 25 374–387. 10.1162/jocn_a_00331 23163412 PMC3932333

[B49] Van Leijenhorst L. Gunther Moor B. Op de Macks Z. A. Rombouts S. A. R. B. Westenberg P. M. Crone E. A. (2010). Adolescent risky decision-making: neurocognitive development of reward and control regions. *Neuroimage* 51 345–355. 10.1016/j.neuroimage.2010.02.038 20188198

[B50] Ward B. D. (2000). *Simultaneous Inference for fMRI Data.* Available at: http://afni.nimh.nih.gov/pub/dist/doc/manual/AlphaSim.pdf

